# Correlation between Protein Sequence Similarity and Crystallization Reagents in the Biological Macromolecule Crystallization Database

**DOI:** 10.3390/ijms13089514

**Published:** 2012-07-27

**Authors:** Hui-Meng Lu, Da-Chuan Yin, Yong-Ming Liu, Wei-Hong Guo, Ren-Bin Zhou

**Affiliations:** 1 Institute of Special Environmental Biophysics, School of Life Sciences, Northwestern Polytechnical University, Xi’an 710072, Shaanxi, China; E-Mails: luhuimeng@nwpu.edu.cn (H.-M.L.); auliuym@mail.nwpu.edu.cn (Y.-M.L.); gwh@nwpu.edu.cn (W.-H.G.); zhourenbin@mail.nwpu.edu.cn (R.-B.Z.); 2 Key Laboratory for Space Bioscience and Biotechnology, Northwestern Polytechnical University, Xi’an 710072, Shaanxi, China

**Keywords:** crystallization reagents, protein sequence similarity, protein crystallization, molecular structure, X-ray crystallography

## Abstract

The protein structural entries grew far slower than the sequence entries. This is partly due to the bottleneck in obtaining diffraction quality protein crystals for structural determination using X-ray crystallography. The first step to achieve protein crystallization is to find out suitable chemical reagents. However, it is not an easy task. Exhausting trial and error tests of numerous combinations of different reagents mixed with the protein solution are usually necessary to screen out the pursuing crystallization conditions. Therefore, any attempts to help find suitable reagents for protein crystallization are helpful. In this paper, an analysis of the relationship between the protein sequence similarity and the crystallization reagents according to the information from the existing databases is presented. We extracted information of reagents and sequences from the Biological Macromolecule Crystallization Database (BMCD) and the Protein Data Bank (PDB) database, classified the proteins into different clusters according to the sequence similarity, and statistically analyzed the relationship between the sequence similarity and the crystallization reagents. The results showed that there is a pronounced positive correlation between them. Therefore, according to the correlation, prediction of feasible chemical reagents that are suitable to be used in crystallization screens for a specific protein is possible.

## 1. Introduction

Protein structure determination is still an important field utilized by many scientists in modern life sciences, as protein structures are the basis of not only protein function studies, but also the structure based drug design. The protein structure entries have increased steadily in Protein Data Bank (PDB) database [1]. More than 88% of protein structures in the PDB were determined by X-ray diffraction (XRD) technique [1]. However, the protein structural entries grew far slower than the sequence entries by one order of magnitude [1,2]. The big gap between the entries of protein structures and sequences was mainly caused by the bottleneck of protein crystallization for XRD technique [3–6].

The difficulty in protein crystallization is mainly due to there being too many parameters, including temperature, pH, crystallization reagents, protein concentration, precipitant concentration, additives, and so on [7,8], that are governing the crystallization process. One of the most difficult obstacles might be choosing appropriate chemical reagents that are capable of crystallizing the target protein, because no one knows which reagent or combination of reagents among so many possibilities can help to crystallize the protein [9–13]. Therefore, a large number of chemical reagents and their different combinations are tested exhaustively hoping for a lucky break (such trial and error testing is called crystallization screening).

To enhance the efficiency in the crystallization screening process, rationally arranging the combinations of chemical reagents for a specific protein might be a solution. However, there is no established method for this purpose. Fortunately, after many years of accumulation, there are some databases consisting of successful crystallization conditions, for example, the Biological Macromolecule Crystallization Database (BMCD) [14], the C6 Web Tool [15] and the XtalBase web-based program [16]. These databases may contain some useful information which can guide us to rationally enhance the efficiency of crystallization screening, and therefore partly avoid this time and energy consuming process.

However, some researchers might argue that there is definitely no relationship between protein type and successful crystallization conditions, although there is no thorough investigation to support this assertion. Now databases like the BMCD contain information of protein types and crystallization conditions, which provide a good opportunity to examine whether there is any relationship between the protein type and the crystallization conditions. Based on this idea, we conducted an investigation about this issue through data mining from the existing databases (the BMCD and the PDB).

The investigation was carried out according to the following guidelines: the protein types can be classified into categories based on their sequence similarity. On the other hand, random groups without sequence similarity from the raw datasets can be established as the control datasets for robust examination. Moreover, the consistency and the differences of the reagent types in each category can be calculated for comparison with those of the overall and the random groups. If the consistency within each category is higher and the difference within each cluster is lower than those of the overall or the random groups, then there should be a correlation between the protein sequence similarity and crystallization reagents. Otherwise the result cannot support the correlation.

## 2. Results and Discussion

### 2.1. Datasets

The 43,406 entries in the BMCD consisting of protein crystallization information were downloaded from the official site of the BMCD [17], and their sequences were downloaded from the PDB database [18]. The entries of the proteins with a length of less than 30 amino acid residues, *i.e*., short peptides, were discarded for this study. The crystallization reagent names of the whole entries were unified. Then the extracted protein entries were clustered into 12,765 groups according to a 100% sequence similarity, and the unified reagents of the same proteins remained. Therefore, the 12,765 entries were used to establish the non-redundant dataset for the next analysis.

**Figure 1:**
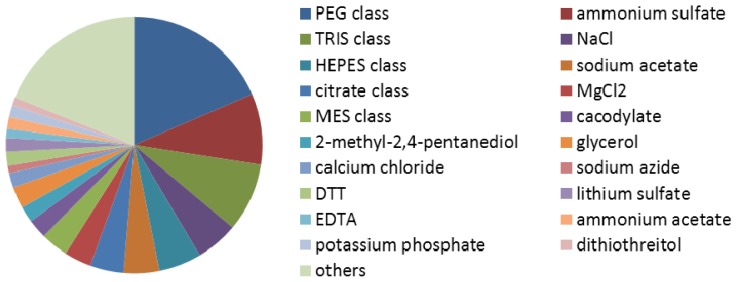
The main crystallization reagents and their frequencies in the non-redundant dataset from the Biological Macromolecule Crystallization Database (BMCD).

The frequency of each kind of crystallization reagent was calculated by Equation (1) from the local non-redundant dataset. The crystallization reagents and their frequencies are shown in Figure 1. Reagents such as PEG class, (NH4)_2_SO_4_, TRIS class, NaCl, HEPES class, sodium acetate, and so on, are clearly dominant in the successful crystallization category.

### 2.2. Establishing the Large Sequence Similarity Clusters (LSSC) Dataset and Random Datasets

Large sequence similarity clusters (LSSC) datasets, including the information of protein sequence similarity and crystallization reagents, were established. Entries of the non-redundant dataset (12,765) were clustered into 5,447 clusters according to more than 30% similarity clustering results by Blastclust software. Then 3,921 entries (belonging to 173 LSSC, the size of which has 10 or more than 10 members per cluster) were extracted to establish the LSSC dataset (named as LSSC30). Then the LSSC40, LSSC50, LSSC60, LSSC70, LSSC80 LSSC90 datasets based on more than 40% to 90% sequence similarity were established in the same way. The entry and cluster numbers of LSSC datasets are shown in Table 1.

Seven random datasets were also established based on the above LSSC datasets by rearranging methods. Therefore, sequences within each cluster in LSSC datasets (LSSC30 to LSSC90) had above 30% to 90% sequence similarity with each other, respectively, but sequences within each group in random datasets were less similar for control analysis.

### 2.3. Statistical Analysis of Reagent Consistency within Each LSSC and Random Group

**Figure 2:**
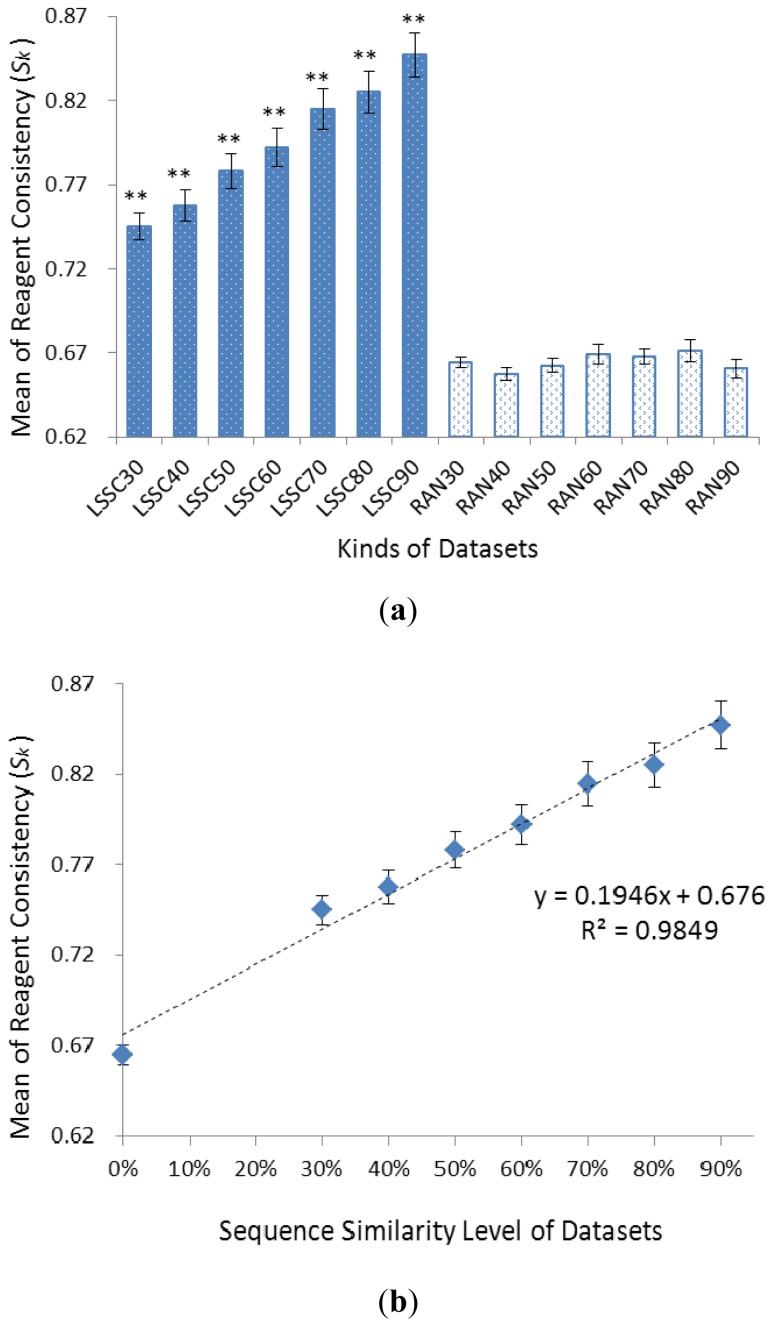
(**a**) The reagent consistency within each LSSC (*S_LSSC_*) and random groups (*S_RAN_*). (Error Bar: standard error of mean; ** *p* < 0.001 of the *t*-test results). (**b**) The reagent consistency against the sequence similarity level of the LSSC and random datasets. (Error bar: standard error of mean; Dashed line: the linear regression line between reagent consistency and sequence similarity.)

The reagent consistency within each LSSC (*S_LSSC_*) and random groups (*S_RAN_*) was both calculated and the result was shown in Figure 2a. The average of *S_LSSC_* values of LSSC30 to LSSC90 datasets were 0.75, 0.76, 0.78, 0.79, 0.81, 0.83 and 0.85, respectively, and the average of *S_RAN_* values of RAN30 to RAN90 datasets were 0.66, 0.66, 0.66, 0.67, 0.67, 0.67 and 0.66, respectively. It can be seen that the seven mean values of *S_LSSC_* (of LSSC30 to LSSC90) were significantly higher than *S_RAN_* (of RAN30 to RAN90), respectively, as proven by two-tailed Student *t*-test (*p* < 0.001). This result verified that most *S_LSSC_* were significantly higher than *S_RAN_*, suggesting that proteins within the same family are more likely crystallized by similar reagents.

Moreover, the linear regression relationship between reagent consistency and sequence similarity was established based on those data (Figure 2b). The linear regression equation was: y = 0.1946× + 0.676, and the correlation exponential of the equation (R^2^) was 0.9849. This result shows the strong positive correlation between reagent consistency and sequence similarity, that is to say, the increase in reagent consistency correlates with the increase in protein sequence similarity.

### 2.4. Statistical Analysis of Reagent Variety between Each LSSC and Random Group

**Figure 3:**
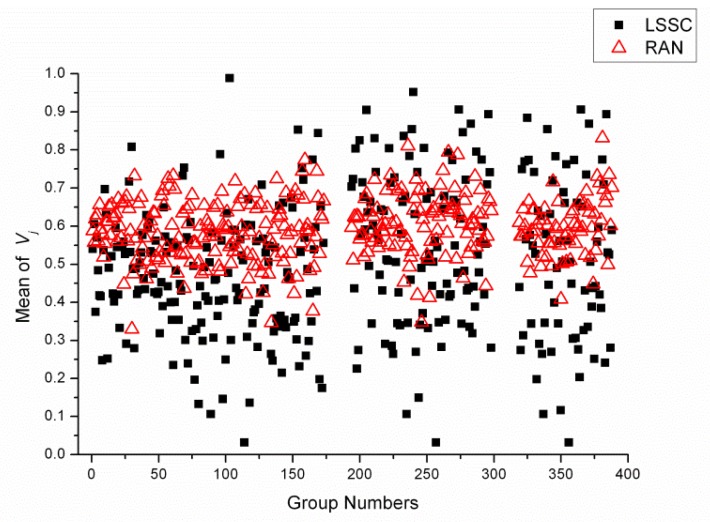
Range of mean *V_j_* values in each cluster in LSSC datasets (from 0.032 to 0.989) was wider than the range in random datasets (form 0.347 to 0.871). (Group numbers: 1~173 belonged to the LSSC30 or RAN30 datasets, 194~298 belonged to the LSSC60 or RAN60 datasets, 319~388 belonged to the LSSC90 or RAN90 datasets; Solid black square: mean *V_j_* in each cluster in LSSC datasets, hollow red triangle: mean of *V_j_* in each group in random datasets.)

The transferred weighted values of reagents (*V_j_*) of each condition were calculated, and the mean *V_j_* values in each cluster in LSSC datasets (LSSC30, LSSC60 and LSSC90) and every group in random datasets (RAN30, RAN60, RAN90) were shown in Figure 3. The range of mean *V_j_* values in LSSC datasets (from 0.032 to 0.989) was wider than the range of random datasets (form 0.347 to 0.871). The wider range of mean *V_j_* values in LSSC datasets showed that the variety of reagents in LSSC was larger than in the random groups. This result can be interpreted to infer that different protein families have different crystallization reagents.

Table 2 shows that most of the variance of *V_j_* (*VAR_j_*) of LSSC clusters were lower than the total variance of *V_j_* (*VAR_total_*). The proportions of lower *VAR_j_* than *VAR_total_* were 76.3%, 77.1% and 75.7% in LSSC datasets, but the proportions were about 54% in the random datasets. This result shows that the variance of transferred weighted values of reagents (*V_j_*) within each LSSC was smaller than the whole variance in the datasets, which also means that the crystallization reagents have a close relationship with the sequence similarity.

Then the differences of *V_j_* within groups were compared with those between groups, by one-way ANOVA method. The resulting *p* values were all less than 0.001 in LSSC datasets, indicating that the *V_j_* differences within each LSSC were significantly smaller than those between different clusters in LSSC30, LSSC60 and LSSC90. In other words, the difference in *V_j_* was significantly affected by the grouping of proteins according to the sequence similarity. On the other hand, the resulting *p* values in three random datasets (RAN30, RAN60, RAN90) were 0.475, 0.716 and 0.962, which were all much larger than 0.05 (*p* > 0.05). This result showed that the *V_j_* differences within each random group were not significantly smaller than those between groups. Therefore, the *V_j_* values of random groups were not relevant to random groups.

In summary, it can be concluded from the above analyses that there was a significant positive correlation between sequence similarity and crystallization reagents, and such correlation was not produced by random events.

Some studies showed that the positive correlation between sequence similarity and crystallization probability drops rapidly below 90% sequence identity, while negative correlation between sequence similarity and the probability of crystallization not being achieved did not drop significantly. This is because negative features impacting crystallization are often conserved in groups of similar sequences [19]. The strong positive correlation between sequence similarity and crystallization reagents achieved in this study can also suggest that some features contained in sequence similarity can also influence the reagent selection of crystallization.

On the other hand, it is known that the structure of a protein molecule is determined by its sequence [20,21], and protein structure affects the crystallization process, because many crystallization influencing factors are determined by their structures, such as solubility, *p*I value, hydrophobicity [22–26]. Hence the result in this study, *i.e.,* the positive correlation between the sequence similarity and crystallization reagents, indicated that the selection of appropriate crystallization reagents is influenced by the molecular structure. This result and previous study of the correlation between protein sequence similarity and X-ray diffraction quality [27] both confirmed that the structure of protein molecules can affect the crystallization process.

## 3. Methods and Experimental Section

### 3.1. Datasets

The Biological Macromolecular Crystallization Database (BMCD) is a publicly available resource, containing information on molecules, crystals and crystallization conditions for macromolecules for which diffraction quality crystals have been obtained [14]. BMCD entries include macromolecule sequence, protein properties and crystallization conditions, which can be downloaded from the internet freely.

Since the structures in the BMCD were determined in different laboratories and/or at different times, the BMCD database contains considerable redundant information for the same proteins. To establish a non-redundant dataset containing information of protein sequences and crystallization conditions, it is necessary to find the unified reagents for the same proteins. Unified reagents, which means all possible reagents for a given protein, were combined from all kinds of reagents appearing for the same proteins, so that each important reagent for the given protein crystallization can be held in reserve. Therefore, the unified reagents are more suitable to represent the requirements of protein crystallization. The current paper will analyze the correlation between the unified reagents and the sequence similarity.

Current release of the BMCD (version 4.03) includes 43,406 crystal entries. Protein crystallization conditions data for the current study were first downloaded from the BMCD (released in May 2012) [17] and data of their amino acid sequences were downloaded from the PDB web site (released in May 2012) [18]. Other data were excluded.

The downloaded data were then screened based on the criterion: the entries left for analysis should consist of proteins of more than 30 amino acid residues. The names of the crystallization reagents were unified, e.g., sodium chloride was converted to NaCl, and all kinds of PEG (such as PEG3350, PEG3000) were converted to “PEG class”. Then the redundant entries were filtered. All of the protein sequences were clustered at 100% sequence similarity level by using the BlastClust program [28,29], so as to filter the protein redundant entries. The unified reagents of the same proteins were left to construct the local non-redundant dataset for the next step of analysis.

### 3.2. Calculating the Frequency of each Kind of Crystallization Reagent

The frequency of each kind of crystallization reagent was calculated from the local non-redundant dataset, to establish the necessity of each reagent for protein crystallization. The frequency of the reagent *i* (*F_i_*) is given by Equation (1).

(1) $$F_{i}=\frac{\sum_{j=1}^{N}x_{ij}}{N}$$

where *x_ij_* = 1 (when reagent *i* appears in condition *j*), *x_ij_* = 0 (when reagent *i* does not appear in the condition *j*), *N*: the total amount of the crystallization condition entries of the non-redundant dataset. For example, if NaCl appeared in 2,194 conditions among the total 12,765 conditions, the frequency of NaCl (*F_NaCl_*) can be calculated as 0.172. Moreover, the 100 highest frequency reagents from the non-redundant dataset were chosen to calculate the similarity of reagents between crystallization conditions, and to convert a reagents combination of a given condition into a transferred weighted value. On the other hand, the *F_i_* values of reagents were used as the weighting factor for calculating analysis.

### 3.3. Clustering by Sequence Similarity to Establish the LSSC Datasets

Sequences of homological protein structures usually have more than 30% similarity [30–32]. Therefore in this study, we adopted the 30% to 90% (10% interval) similarity levels as thresholds to categorize the proteins from the local non-redundant dataset into seven sequence similarity cluster (SSC) datasets by BlastClust program. Thus, for example, proteins within every clusters of SSC30 had at least 30% sequence similarity.

Some of the SSC may have less than 10 members, which means that those protein families have been seldom crystallized, and may not be able to provide enough information for our study. Therefore, we built the large sequence similarity clusters (LSSC) datasets from SSC clusters with 10 or more than 10 entries. These seven LSSC datasets (named as LSSC30, LSSC40, …, LSSC90, respectively) were constructed and used for further analysis to check the relationship between the reagents and the sequence similarity.

### 3.4. Establishing the Random Datasets from the LSSC Datasets for Robust Analysis

To check the reliability of the analysis results, we established seven random datasets (named as RAN30, RAN40, …, RAN90, respectively) based on the seven LSSC datasets and did the same approaches on them (as those on the LSSC datasets) to analyze the relationship between the reagents and the random groups. The procedures were as follows: all entries of LSSC datasets were randomly rearranged into groups (the amounts of group number and group size are both same as those of LSSC datasets). Rearranged datasets had the same number of entries grouped randomly against LSSC datasets and did not contain the repeated entries. To check whether the reagents were affected by random grouping or not, we performed the same statistical analysis process on the random datasets. The results of the analysis between random datasets and LSSC datasets were compared, to find out whether the correlation between the reagents and the sequence similarity is a significant rule or just a random phenomenon.

### 3.5. Statistical Analysis of Reagent Consistency within Each Group

The similarity of reagent kinds within each LSSC (*S_LSSC_*) was assessed and compared with the similarity of random groups (*S_RAN_*). The similarity of reagent kinds of crystallization conditions in group *k* (*S_k_*) of LSSC or random datasets is defined by Equation (2):

(2) $$S_{k}=\frac{\sum_{i=1}^{m}\left(S_{ik}F_{i}\right)}{\sum_{i=1}^{m}\left(F_{i}\right)}$$

where *n* is the number of crystallization condition entries in group *k*, *m* is the number of the considered high frequency reagent number (*m* = 100, in this paper), *s_ik_* is similarity of reagent *i* in group *k*, which is defined as: ${\text{s}}_{ik}=2\left|\frac{\sum_{j=1}^{n}x_{ijk}}{n}-0.5\right|$. (*s_ik_* = 0 means the lowest similarity of reagent *i*, when reagent *i* appeared at just half conditions in group *k; s_ik_* = 1 means the highest similarity of reagent *i*, when reagent *i* appeared at either all or none conditions in group *k*.) *x_ijk_* and *F_i_* is the value of reagent *i* of condition *j* in group *k* and the frequency of the reagent *i* in the whole dataset, respectively, as already assigned in Equation (1). For example, if group *k* has 50 entries, which mainly 100 reagents appeared 46, 0, 8 … times and had 0.593, 0.286, 0.276 … frequencies in the whole dataset respectively, the similarity of reagents in group *k* (*S_k_*) can be calculated as: $\left(2\times \left|\frac{46}{50}-0.5\right|\times 0.593+2\times \left|\frac{0}{50}-0.5\right|\times 0.286+2\times \left|\frac{8}{50}-0.5\right|\times 0.276+\cdots )/(0.593+0.286+0.276+\cdots \right)$.

Then the seven pairs of average values of reagent consistency of clusters or groups between LSSC dataset and random datasets (Means of *S_LSSC_* or *S_RAN_*) could be compared and tested for significance of difference respectively, with use of Student *t*-test.

### 3.6. Statistical Analysis of Reagent Variety between Groups

We used variance comparison and one-way ANOVA to analyze the reagent variety between each LSSC or random groups of the datasets, so as to check the correlation between the reagents and the sequence similarity from another point of view. The transferred weighted value of reagents of each condition for ANOVA analysis was assigned as the sum of the products of each reagent value and its frequency, as given by Equation (3) for condition *j*:

(3) $$V_{j}=\sum_{i=1}^{m}\left(x_{ij}F_{i}\right)$$

where *m* is the number of 100 high frequency reagent types, *V_j_* is the transferred weighted value of reagents in condition *j*, *x_ij_* is the value of reagent *i* in condition *j*, as already assigned in Equation (1). For example, if just NaCl and MgCl_2_ appeared in condition *j*, the transferred weighted value of reagents in condition *j* (*V_j_*) can be calculated as 0.281 (*i.e.*, 1 × 0.172 + 1 × 0.109).

The variance of *V_j_* (*VAR_j_*) of each clusters or groups about LSSC and random datasets was calculated and compared with the total variance of *V_j_* (*VAR_total_*) about LSSC and random datasets, respectively. If most of *VAR_j_* values were lower than *VAR_total_*, it will mean that reagent differences within groups are lower than the whole difference of the dataset. Moreover, the *V_j_* values of LSSC and random datasets were statistically analyzed by one-way ANOVA method to check whether the reagent differences between groups are significantly larger than those within groups. It will mean that reagent differences between groups are significantly larger than those within groups, if the resulting *p* value is less than 0.01.

**Figure 4:**
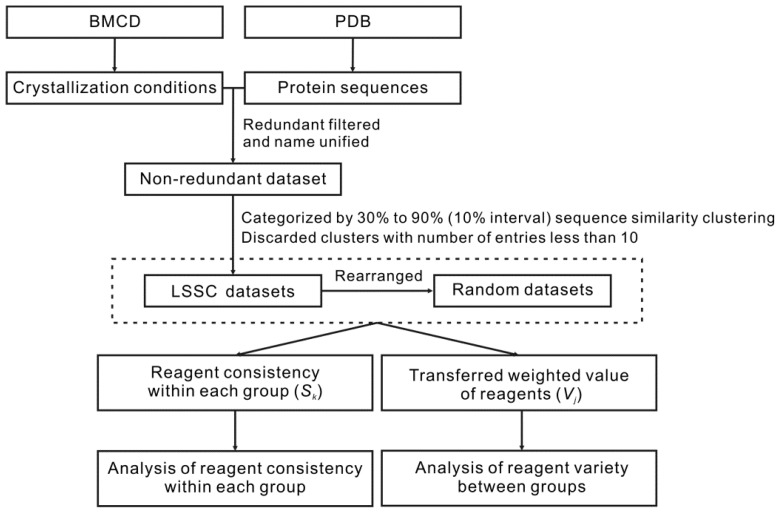
The analysis strategy and process of this work.

The analysis strategy of this work is shown in Figure 4.

## 4. Conclusions

In this investigation, information on crystallization reagents and the sequence of proteins was extracted from the BMCD and the PDB databases. Then the relationship between the sequence similarity and the unified reagents was statistically analyzed. The results showed that protein crystallization reagents were significantly positively correlated with protein sequence similarity. The robust estimation results showed that correlation was not caused by random events. The results also indicated that protein structure determined by sequence would affect the crystallization process.

The current work further indicated that rationally selecting crystallization reagents and the design of a new crystallization screening kit for crystallizing a specific protein is feasible. Or according to the correlation, it is possible to predict preferable crystallization reagents for a new protein, and thereby select the most suitable screening kit from those commercially available.

## Figures and Tables

**Table 1:** The numbers of clusters and entries of the large sequence similarity clusters (LSSC) datasets.

LSSC datasets	Number of clusters	Amount of entries	Average size of each cluster
LSSC30	173	3,921	22.7
LSSC40	144	3,006	20.9
LSSC50	122	2,433	19.9
LSSC60	105	2,068	19.7
LSSC70	87	1,757	20.2
LSSC80	81	1,586	19.6
LSSC90	70	1,340	19.1

**Table 2:** Comparison of *V_j_* *variance (VAR*_j_) between LSSC and Random datasets.

Datasets	*VAR_total_*	Group number under *VAR_total_*	Group size	Proportion of lower *VAR_j_* than *VAR_total_*
LSSC30	0.109	132	173	76.3%
LSSC60	0.111	81	105	77.1%
LSSC90	0.097	53	70	75.7%
RAN30	0.099	93	173	53.8%
RAN60	0.101	58	105	55.2%
RAN90	0.098	38	70	54.3%
